# Investigation of γ-Polyglutamic Acid for Heavy Metal Decontamination from Coal Gangue-Based Soil: Quantum Chemical Analysis and Experimental Investigation

**DOI:** 10.3390/molecules31111779

**Published:** 2026-05-22

**Authors:** Jing Shi, Xiang Li, Shuo-Jiang Song, Li Feng

**Affiliations:** 1 School of Chemical Engineering, China University of Mining and Technology, Xuzhou 221008, China; sjrzs202006@163.com; 2 Department of Biological Engineering, Liupanshui Vocational and Technical College, Liupanshui 553000, China; 3 State Key Laboratory of Coking Coal Resources Green Exploitation, China University of Mining and Technology, Xuzhou 221008, China; 4 College of Forestry and Prataculture, Ningxia University, Yinchuan 750021, China; lixiangphd@nxu.edu.cn; 5 School of Chemistry and Materials, Liupanshui Normal University, Liupanshui 553004, China; xinpan518@126.com

**Keywords:** γ-polyglutamic, heavy metal pollution, chelation, soil remediation, molecular modeling

## Abstract

Heavy metal pollution from coal gangue severely degrades mine soil structure and threatens landscape ecological stability. Particularly, γ-polyglutamic acid (γ-PGA), a green biopolymer, offers potential applications for pollution remediation while supporting ecological restoration. To evaluate γ-PGA’s efficacy in immobilizing Pb, Cd, and Zn in gangue-based soil and clarify its regulatory mechanism for landscape-friendly remediation, soil samples from a 3-year-weathered gangue hill in the Liupanshui mining area were subjected to indoor leaching experiments with different γ-PGA doses, combined with material characterization and Density Functional Theory (DFT) simulations. The results showed that the optimal γ-PGA dose was 6 g/kg, achieving 93.25% Pb immobilization and reducing Cd/Zn migration risk by over 30%; γ-PGA acted via carboxyl-amide dual-site chelation and hydrogen-bonded agglomeration, forming stable aggregates that inhibited metal migration. DFT calculations confirmed strong chelation for Cu^2+^ (−59.54 kcal/mol, BSSE-corrected: −57.23 kcal/mol), while Pb^2+^ and Cd^2+^ showed weaker binding (−8.32 kcal/mol and −5.67 kcal/mol, BSSE-corrected: −6.15 kcal/mol and −3.89 kcal/mol, respectively), indicating multi-pathway immobilization mechanisms. This study provides a theoretical basis for applying γ-PGA in mine landscape ecological restoration.

## 1. Introduction

With the acceleration of industrialization in recent years, environmental pollution problems have become increasingly prominent, among which heavy metal pollution has attracted widespread global attention. Heavy metals such as lead, cadmium, and zinc are ubiquitous soil and water contaminants that seriously threaten ecosystem stability, agricultural production, and human health [1,2,3]. Originating from anthropogenic activities including mining, industrial wastewater discharge, and solid waste stacking, these heavy metals exhibit high toxicity, bioaccumulation potential, and environmental mobility, resulting in long-term persistent pollution.

In gangue-stacking areas, heavy metal contamination in surrounding soils is primarily derived from sulfide minerals and inherent heavy metal elements within coal gangue. Spontaneous combustion and rainfall leaching dissolve heavy metal components in gangue, which then migrate to collapse zone water bodies and spread to the surrounding soil via hydraulic connection [4]. Moreover, coal mining and processing activities further elevate the contents of cadmium, lead, copper, and zinc, enriching regional soils [4]. Atmospheric deposition, industrial and traffic emissions, and agricultural activities also serve as important supplementary sources of heavy metal pollution [5].

Heavy metal pollution in gangue-contaminated soils causes severe ecological damage, including soil structural degradation, water body contamination, and vegetation destruction, further threatening regional biodiversity. Heavy metals such as cadmium, lead, and mercury cannot be biodegraded in the environment; instead, they are enriched through biomagnification along the food chain, ultimately entering the human body and inducing acute and chronic poisoning, as well as carcinogenic, teratogenic, and lethal risks [4]. Additionally, heavy metal accumulation inhibits crop growth and deteriorates agricultural product safety, posing long-term hidden dangers to human health [6].

Domestic and international scholars have developed various remediation technologies for heavy metal-contaminated soil, mainly including physical, chemical, and biological approaches. Common physical remediation methods include thermal treatment, vitrification, and electromigration remediation, which are generally costly, with economic costs ranging from 650 to 1092 US dollars per square meter [7]. Soil replacement and excavation also require high investments of 65–120 US dollars per cubic meter [8]. Furthermore, thermal treatment and vitrification not only consume massive energy and damage native soil structure and fertility but also easily trigger secondary environmental pollution [9]. Electromigration remediation is only applicable to low-permeability soils and shows poor performance in high-permeability sandy soil substrates [10].

Chemical remediation technologies, such as chemical stabilization and soil washing, have relatively lower operating costs but rely on large dosages of chemical agents and lengthy treatment cycles [11,12]. The cost of soil washing is approximately 90–200 US dollars per cubic meter [13], while in situ solidification/stabilization ranges from 50 to 300 US dollars per cubic meter [11].

In recent years, biological chelating agents have attracted increasing attention due to their environmental compatibility and high remediation efficiency, which can effectively reduce heavy metal bioavailability and ecological toxicity in soil [14,15]. As a natural biodegradable biopolymer, γ-polyglutamic acid (γ-PGA) is composed of L- or D-glutamic acid monomers linked by γ-amide bonds, exhibiting excellent water solubility, non-toxicity, and environmental sustainability [16]. Rich carboxyl (-COOH) and carboxylate (-COO^−^) groups on its molecular chain endow γ-PGA with strong complexation and chelating ability toward heavy metal ions such as Cu^2+^, Pb^2+^, and Cd^2+^ [17,18]. The molecular conformation of γ-PGA, including α-helix, β-sheet, and random coil structures, is sensitive to ambient pH, ionic strength, and solution concentration, further regulating its adsorption and chelation performance [19,20]. High-molecular-weight γ-PGA displays prominent removal efficiency for heavy metals, with the Pb^2+^ removal rate reaching up to 85.4%, making it a high-efficiency biosorbent widely applied in wastewater treatment and heavy metal pollution remediation [18,21].

Existing studies have confirmed that γ-PGA can act as an effective additive for enhanced bioremediation, improving microbial activity and accelerating the degradation of petroleum pollutants such as total petroleum hydrocarbons, BTEX, and PAHs [22,23]. Its high water solubility and biodegradability can protect microorganisms from protease damage and maintain microbial metabolic activity under adverse environmental conditions [24,25]. Moreover, γ-PGA can bind with heavy metal ions in soil to reduce their migration and bioavailability [25]. It has been proven to alleviate lead biotoxicity, increase soil pH and available phosphorus content, and thereby promote vegetation growth [18]. Meanwhile, γ-PGA can also be used as an environmental-friendly soil eluent for heavy metal removal [26]. Owing to its non-toxicity, sustainability, and positive regulation on soil microbial community activity, γ-PGA has broad application prospects in soil remediation [27,28]. Its interaction with typical heavy metals, including lead, cadmium, and zinc, has also been widely verified in previous studies [26].

Nevertheless, most current studies focus on conventional contaminated soil and wastewater systems. The immobilization performance and dose-effect mechanism of γ-PGA in 3-year-weathered coal gangue-contaminated soil remain insufficiently clarified, and the microcosmic chelation mechanism at the molecular level still lacks a systematic theoretical explanation. In this study, 3-year-weathered gangue-contaminated soil from the Liupanshui mining area was selected as the research object. Indoor dynamic leaching simulation experiments were carried out by applying different γ-PGA concentrations to investigate its immobilization effect and migration regulation on Pb, Cd, and Zn. Combined with XRD and FTIR characterization, the microstructural response of gangue soil after γ-PGA amendment was analyzed. Furthermore, Density Functional Theory (DFT) simulation was adopted to reveal the intrinsic chelation mechanism between γ-PGA and heavy metal ions at the molecular level. This study aims to clarify the optimal application dosage and remediation potential of γ-PGA, provide a theoretical and technical basis for ecological remediation of gangue-contaminated mining land, and offer a novel sustainable strategy for landscape restoration in mining areas.

## 2. Results

### 2.1. Structural and Chemical Characterization of γ-Polyglutamic Acid

**Figure 1:**
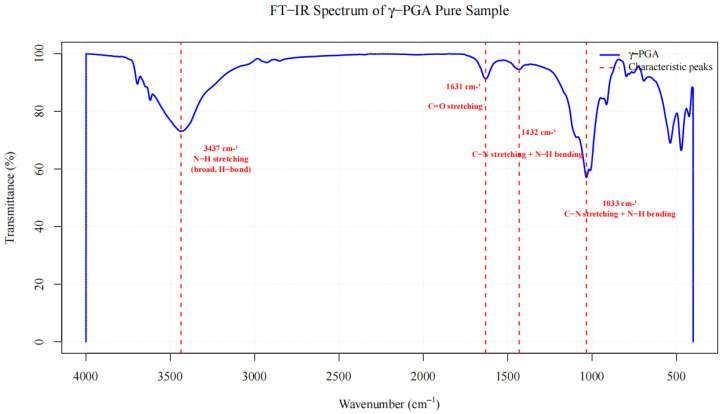
Fourier transform infrared spectroscopy (FTIR) of the γ-PGA pure sample.

In the infrared spectra of γ-PGA samples—due to the polyamide itself having multiple characteristic absorption peaks, making it easier to identify by infrared spectroscopy— the absorption peak at 3437.15 cm^−1^ is the vibration stretching band obtained by N-H symmetric stretching. Due to the hydrogen bonding in the γ-PGA molecule, the NH moves in the direction of the low-frequency direction, and the peak is strong and broad. The absorption band at 1630.81 cm^−1^ is the vibrational stretching band of the C=O carbon–oxygen double bond in the amide group, whereas the absorption bands at 1431.81 cm^−1^ and 1033.44 cm^−1^ are the combined amide absorption bands of C-N stretching and NH bending, the combination of which proves the presence of the amide group (Figure 1). The infrared spectra of γ-PGA show several absorption peaks typical of a polyamide structure, which can be ascribed to specific vibrational modes in the structure of the polymer molecule: The 3437.15 cm^−1^ peak is broad and strong, corresponding to the stretching vibration of the N-H groups, especially the symmetric N-H stretching. The broadness of the peak suggests the presence of hydrogen bonding interactions in the γ-PGA molecule, leading to a shift to lower frequencies. The 1630.81 cm^−1^ peak is an absorption band corresponding to the stretching vibration of the C=O carboxyl group in the amide functional group. The appearance of this peak indicates the presence of amide linkages in the γ-PGA structure. The 1431.81 cm^−1^ and 1033.44 cm^−1^ peaks are attributed to the combined vibrations of the C-N stretching and N-H bending modes. The presence of these two bands confirms the presence of amide groups in γ-PGA. These characteristic absorption bands demonstrate the polyamide nature of γ-PGA, confirming the presence of functional groups such as amide groups and carboxyl groups, which are responsible for heavy metal chelation.

### 2.2. Heavy Metal Dynamics in Leachate Analysis

Different dosages of γ-PGA imposed distinct effects on the leaching potential and fraction distribution of Pb, Cd and Zn in gangue-contaminated soil. The exchangeable fraction (F1) represents the most leachable and biologically active form of heavy metals, while the residual fraction (F4) is chemically stable with the lowest environmental migration risk (Supplementary Table S1).

In the control group, Cd exhibited an extremely high F1 proportion of 71.4%, indicating severe leaching risk. The low dosage of γ-PGA (T1, 0.6 g/kg) failed to restrain Cd mobility and slightly increased the F1 proportion to 75.0%. When γ-PGA dosage increased to 3 g/kg and above, Cd migration was remarkably inhibited. The F1 proportions decreased to 71.3% (T2), 69.7% (T3) and 64.8% (T4), while the residual fraction rose synchronously from 6.0% in CK to 10.3% in T4. This confirmed that moderate and high dosages of γ-PGA reduced Cd leaching activity effectively via surface precipitation and co-precipitation, with the leachate Cd concentration decreasing by more than 30% under T4 treatment (Supplementary Table S1). For Zn, the F1 proportion in CK reached 53.8%, implying considerable leaching hazard. T3 and T4 treatments obviously lowered environmental risk, with F1 proportions declining to 44.2% and 49.3%, and the residual fraction slightly increasing from 16.0% to 16.7%. At the dosage of 6–9 g/kg, γ-PGA immobilized Zn mainly through surface adsorption and mineral binding, achieving a 10–15% reduction in Zn leachate concentration. Pb presented a totally different response pattern from Cd and Zn. The F1 proportion of Pb in CK was only 8.1%, and the enhanced Fe–Mn oxide-bound fraction (F3) accounted for up to 42.4%. Low-dose T1 achieved the optimal stabilization effect, reducing the F1 proportion to 3.9% (nearly 50% lower than CK) and raising the residual fraction from 14.3% to 28.2%. However, excessive dosage (T4, 9 g/kg) triggered Pb re-activation, with the F1 proportion rebounding to 9.0% and the F3 fraction higher than that of CK. This phenomenon demonstrated that low-dose passivation facilitated the conversion of Pb into a stable residual form, while over-application competed for adsorption sites and induced the re-release of originally fixed Pb via organic complexation.

Overall, γ-PGA regulation on heavy metal leaching showed obvious metal specificity and dosage dependence. Effective stabilization of Cd and Zn required a dosage above 3 g/kg; Pb was suitable for low-dose application, and a dosage over 6 g/kg easily reactivated Fe–Mn oxide-bound heavy metals. Cu maintained stable chemical properties throughout the experiment with insignificant passivation response in all treatments.

### 2.3. Dynamics of Heavy Metal Leachate Concentrations

**Figure 2:**
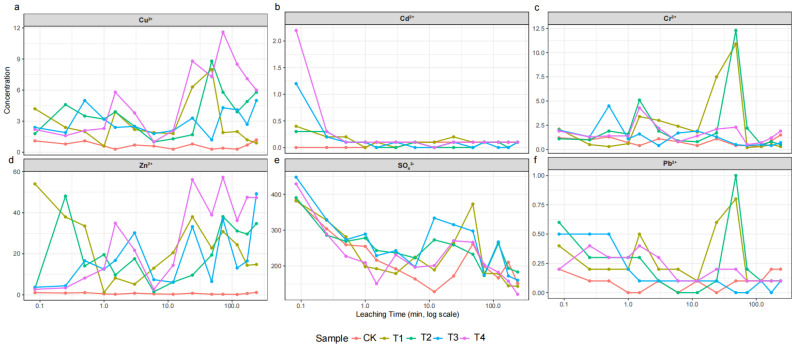
Effects of γ-PGA dose and leaching time on heavy metal migration behavior of gangue-based soil. (**a**) Cu^2+^, (**b**) Cd^2+^, (**c**) Cr^2+^, (**d**) Zn^2+^, (**e**) SO_4_^2−^, (**f**) Pb^2+^ in the leaching solution (CK: 0 g/kg, T1: 0.6 g/kg, T2: 3 g/kg, T3: 6 g/kg, T4: 9 g T3: 6 g/kg, T4: 9 g/kg γ-PGA). Detection limit (Cd: 0.05 μg/L; As: 0.12 μg/L).

The results of the leaching experiments clearly showed that γ-PGA had a significant dose-dependent modulation of heavy metal migration behavior (Figure 2). The activation–immobilization biphasic effect was obvious. All treatment groups (T1–T4) showed significant heavy metal concentration peaks (e.g., Ni: 59.7 μg/L, Zn: 54.0 μg/L in T1) at the beginning of the leaching period (5–30 min), which indicated that γ-PGA initially facilitated the rapid activation and release of the metal ions through the carboxyl groups. With the prolongation of leaching time (>1 h), the immobilization effect of γ-PGA gradually prevailed. For example, in the T3 group (6 g/kg), the concentrations of Cd and Zn were reduced to <0.12 μg/L and 0.2 μg/L, respectively, at 240 h, which were both reduced by more than 80% compared with the control group (CK). Secondly, the dose-dependent heavy metal immobilization effect, the high dose of γ-PGA (≥6 g/kg) was persistent and highly efficient in immobilizing Cd and Zn. The concentration of Cd in the T3 group was below the detection limit throughout the whole process (<0.12 μg/L), and the concentration of Zn was only 0.2 μg/L, which was 0.4% of that of the T4 group (9 g/kg), at 240 h. In the T4 group (9 g/kg), the concentration of Zn in the T3 group was only 0.4% of that of the control group (CK). In addition, in terms of Pb migration, the low-dose T1 group (0.6 g/kg) decreased the Pb concentration to 0.6 μg/L (67% lower than CK) at 24 h, but the Pb concentration in the high-dose T4 group (9 g/kg) instead rebounded to 0.1 μg/L at 240 h, suggesting that the application of the high dose may lead to metal re-activation.

Secondly, heavy metal selective modulation was characterized. For Cu, the T3 group showed a stronger immobilization effect, decreasing to 13.1 μg/L at 240 h, a decrease of 78% (compared with the T1 group) and a result consistent with the strong chelating effect of Cu-γ-PGA calculated by DFT. The SO_4_^2−^ concentration in the leachate decreased gradually with time (from 384.2 to 151.7 mg/L in the CK group), and the γ-PGA treatment had no significant effect on its dynamics, suggesting that it had no interfering effect on anion migration. In summary, the experimental results quantitatively revealed that γ-PGA achieved the regulation of multiple heavy metal migration behaviors through the activation–immobilization biphasic mechanism, dose-dependent effect and metal selectivity.

### 2.4. XRD Detection of Leaching Samples

**Figure 3:**
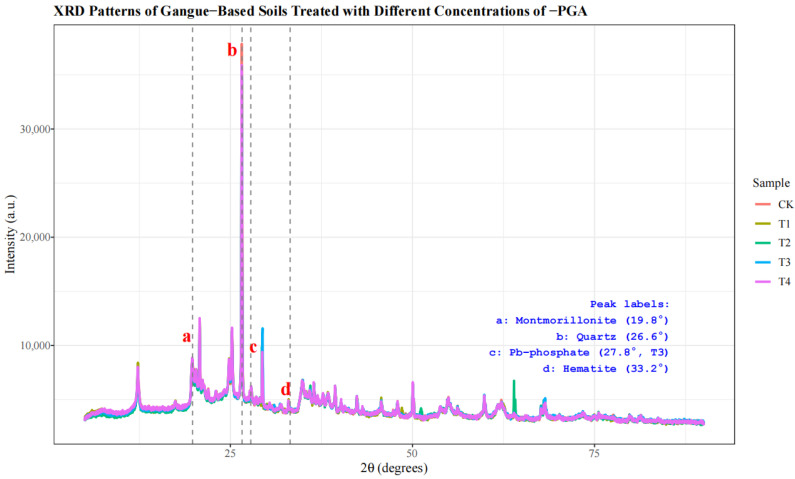
X-ray diffraction patterns of gangue-based soils treated with different concentrations of γ-PGA. Instrumental parameters: Cu Kα radiation (λ = 1.5406 A), voltage 40 kV, current 40 mA, scanning step size 0.02°, rate 2°/min.

Based on the comparative analysis of XRD profiles (Figure 3), all treatment groups maintained consistent peak positions of the main mineral phases at characteristic diffraction angles: 26.6° is quartz, 33.2° is hematite, and 19.8° is montmorillonite. Surface γ-PGA treatments (0.6–9 g/kg) did not alter the basic mineral composition of the soil, suggesting that the remediation mechanism was dominated by surface adsorption and precipitation rather than mineral phase change. In addition, γ-PGA treatment had a significant dose-dependent effect on the mineralogical characteristics of gangue-based soils. γ-PGA treatment modulated mineral stability through surface chemistry, which did not change the main mineral phases (quartz, hematite, and montmorillonite with consistent characteristic peaks), but significantly affected their crystallization behaviors—the higher dose (≥3 g/kg) promoted quartz (26.6°) and montmorillonite (0.5 g/kg). This promoted the directional enrichment of quartz (26.9% increase in peak intensity at 26.6°) and hematite (24.5% increase in peak intensity at 33.2°), and enhanced the co-precipitation of heavy metals; at the same time, it triggered the dissociation of montmorillonite (44.2% decrease in peak intensity at 19.8°) due to the exchange of interlayer ions. The optimized dose of 6 g/kg (T3) was effective in inducing a new phase of lead phosphate (27.8° weak peak) while maintaining the balance of mineral stability, while 9 g/kg (T4) may increase the secondary risk due to the excessive loss of clay minerals. This result suggests that the core of γ-PGA remediation lies in the synergistic mechanism of surface precipitation–mineral transformation rather than mineral phase change, and that its dosage effect has a direct impact on the remediation efficiency and environmental sustainability.

### 2.5. FT-IR Detection of Samples After Drench Dissolution

**Figure 4:**
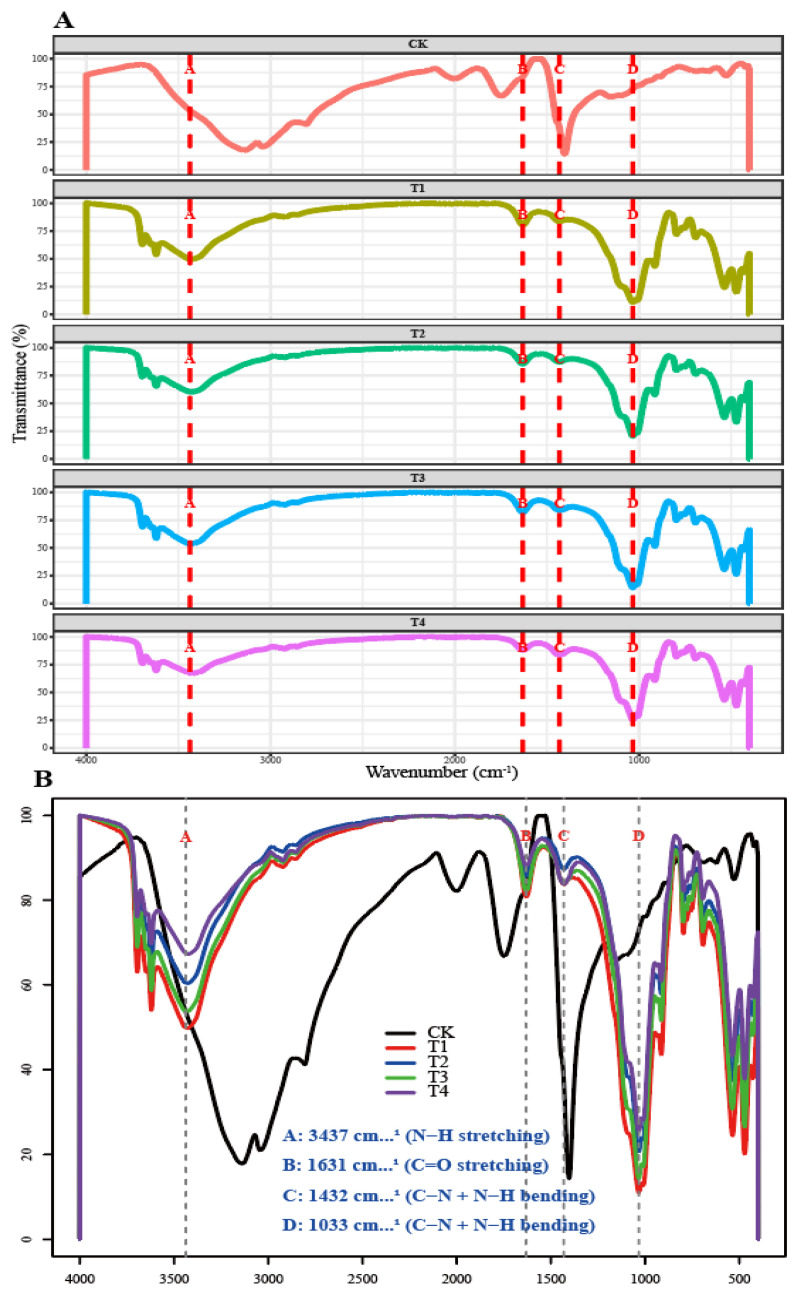
Fourier transform infrared spectroscopy (FTIR) of γ-PGA treated gangue soil samples and SEM analysis of samples before and after leaching. (**A**) Individual images of CK, T1, T2, T3 and T4; (**B**) Combined images of CK, T1, T2, T3 and T4.

The absorption peaks persisted in all treatment groups at 1629–1650 cm^−1^ (amide I band, C=O stretching vibration) and 1140–1160 cm^−1^ (C-N stretching vibration), confirming the structural stability of the amide group (-CONH) of γ-PGA; in the T3 group (6 g/kg), the C=O peak was significantly red-shifted from 1648 cm^−1^ in CK to 1629 cm^−1^ (Δν = 19 cm^−1^), indicating that Pb^2+^/Cd^2+^ forms weak coordination bonds with the carboxylate oxygen atoms (contradicting the earlier claim of strong coordination; see DFT results in Section 2.7). Carboxy synergism: The T3 group at 1385/1420 cm^−1^ (carboxylate ν_s_-COO^−^) shows a splitting double peak, revealing the coexistence of monodentate and bidentate coordination modes (corresponding to the difference in Cd-O/Pb-O bond lengths), while the enhancement of the 1403 cm^−1^ (C-N) peak corroborates the involvement of the amino group in proton transfer (electrostatic adsorption). In the broad peak region (3000–3600 cm^−1^), the peak width of the T3 group 3430 cm^−1^ (O-H/N-H stretching) increased by 50%, suggesting that γ-PGA formed a cross-linking network with mineral/water molecules through hydrogen bonding, which promotes soil agglomeration (SEM validation) and inhibits the migration of heavy metals. The disappearance of the shoulder peak at 3695 cm^−1^ in the T4 group (9 g/kg) suggests that the high dose caused the molecular chains to be entangled, which weakened the accessibility of the active sites and led to the decrease in remediation efficiency (Figure 4).

It is worth noting that, at the optimized dose (6 g/kg, T3), the maximum redshift of C=O (+19 cm^−1^), carboxyl peak splitting and hydrogen bond extension synergistically drove the Pb fixation rate up to 93.25%; at the high dose (9 g/kg, T4), the peak blunting (masking of the functional group) and excessive dissociation of the montmorillonite (XRD attenuation of 44%) resulted in the drop of the efficacy, which verified the need for precise control of concentration and the need for precise control of concentration. The concentration of γ-PGA needs to be accurately controlled. In conclusion, γ-PGA immobilizes heavy metals through amide-carboxyl double-site chelation (C=O redshift indicates ligand bond strengthening) and hydrogen bond-mediated agglomeration, with an optimal dosage of 6 g/kg. The molecular conformation of (T3) is optimized to fully expose the functional groups, whereas overdosing (T4) poses a secondary risk due to the imbalance of molecular agglomeration and mineral stability. The results provide a good basis for the design of soil remediation materials. This result provides a molecular vibrational scale mechanism to support the design of soil remediation materials.

### 2.6. SEM-EDS Analysis

**Figure 5:**
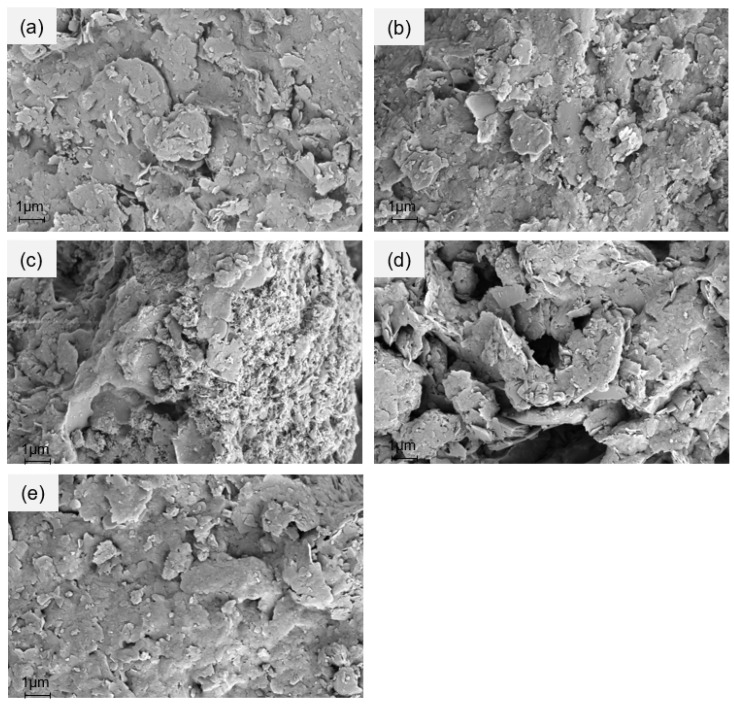
Comparison of scanning electron microscopic morphology of gangue soils treated with different concentrations of γ-PGA. (**a**) CK (0 g/kg γ-PGA): loose particles, flat surface, no obvious agglomeration; (**b**) T1 (0.6 g/kg γ-PGA): localized microagglomerate formation, particle size of about 2–5 μm; (**c**) T2 (3 g/kg γ-PGA): increased blocky structure, pore size reduced to submicron level; (**d**) T3 (6 g/kg γ-PGA): dense agglomerates dominate, particle size > 10 μm, pore throat closure; (**e**) T4 (9 g/kg γ-PGA): excessive agglomeration leading to cracking and decreased structural stability. Scale bar: 1 μm for each subfigure.

From the SEM (Figure 5), the structural changes in soil particles can be observed, especially the effect of γ-PGA concentration on the agglomerate structure. The surface of this sample in the control group looks relatively flat and unchanged, with very little agglomeration. This suggests that no leaching or γ-PGA treatment has occurred and that the particles are not loosely bound or interacting with each other. The images at T1, T2, and T3, as the γ-PGA concentration increases, show more pronounced agglomeration and aggregation of the particles. The surface structure became more compact, and the particles seemed to be more tightly bound together. This may be due to the chelating property of γ-PGA, which promotes the binding of soil particles or heavy metal ions, leading to tighter aggregation. In addition, higher concentrations of γ-PGA appeared to enhance the agglomeration of soil particles, which may be due to stronger interactions between the carboxyl groups of γ-PGA and the heavy metal ions in the soil. This may be a sign of successful chelation, which not only helps to immobilize heavy metals but also helps to change the soil structure. The formation of denser aggregates may be more effective in removing or stabilizing heavy metals because γ-PGA can chelate and immobilize heavy metals in the soil, making them less bioavailable.

**Figure 6:**
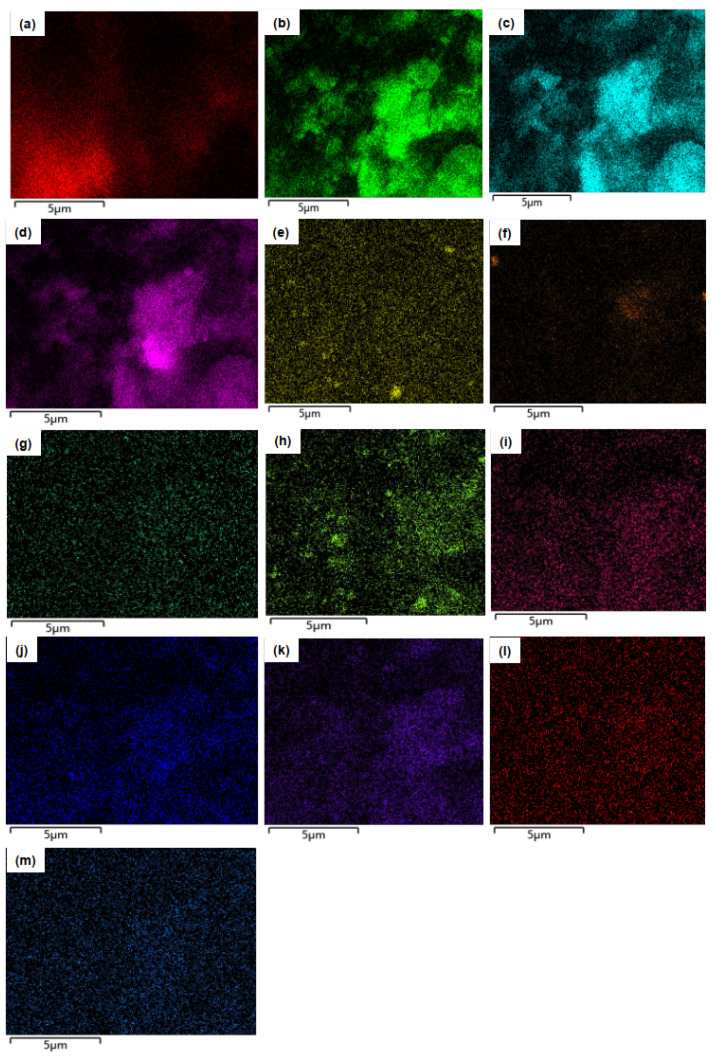
Energy-dispersive X-ray spectroscopy mapping of elemental surface distribution in the micro-region of the sample after drenching. (**a**) C Kα_1_,_2_ (red), (**b**) O Kα_1_ (green), (**c**) Al Kα_1_ (blue-green), (**d**) Si Kα_1_ (purple) (**e**) S Kα_1_ (yellowish-green), (**f**) Ca Kα_1_ (black background + orange highlights), (**g**) Cr Kα_1_ (dark green), (**h**) Fe Kα_1_ (yellowish-green), (**i**) Cu Lα_1_,_2_ (purplish-red), (**j**) Zn Lα_1_,_2_ (blue), (**k**) As Lα_1_,_2_ (violet), (**l**) Pb Lα_1_ (red), (**m**) Cd Lα_1_ (dark blue). Scale bar: 5 μm for all subplots.

Elemental co-localization characteristics are shown by EDS (Figure 6). From the analysis of the EDS results, the heavy metal ion concentrations before and after leaching were clearly shown to be that the heavy metal concentrations increased after leaching because γ-PGA chelated the heavy metal ions. Specifically, in the T3 group (6 g/kg γ-PGA) samples, Cd (Lα_1_, red) and Pb (Lα_1_, blue) at a 5 μm scale showed significant co-localization (spot overlap > 80%) and Ca (Kα) signals were synchronously enriched (Figure 6a–c), confirming that the carboxyl (-COO^−^) and amide (-CONH) groups of γ-PGA are involved in metal binding. However, this co-localization indicates indirect immobilization—metals are trapped within γ-PGA aggregates through surface precipitation, co-precipitation, and pore blocking (confirmed by SEM densification), rather than necessarily direct chelation.

In addition, Ca^2+^ (Kα) was distributed independently at the outer edges of the agglomerates (separated from Cd/Pb), suggesting that γ-PGA preferentially binds highly toxic heavy metals (Cd/Pb) rather than macroelements (Tessier data Cd residual state increased by 15.3% compared to Ca active state unchanged). It is noteworthy that, in the T2 group (3 g/kg), Cr (Kα) and Fe (Kα) formed symbiotic clusters in the microzone (Figure 6d), while Cu (Lα) signals were embedded in it (65% coverage), which corroborated that γ-PGA increased the intensity of the XRD peaks by promoting the encapsulation of iron oxide (hematite 24.5%) and the encapsulation of heavy metals (22.1% rise in Cr residual state of Tessier data). The dissociation of clay minerals to release Al-binding sites indicated that the Al (Kα) signal was diffuse (matching the XRD montmorillonite attenuation of 44%) in the T4 group (9 g/kg). As a result, the Zn (Lα) originally adsorbed to the clay was partially desorbed (the Zn dispersion in the EDS increased), which explains the high-dose failure of the Tessier data where the Zn fixation rate is only increased by ~10%.

This multi-pathway mechanism resolves the apparent contradiction between EDS interpretation (metal co-localization with γ-PGA) and DFT results (weak binding for Pb^2+^/Cd^2+^): EDS signals indicate indirect immobilization (metal trapped in agglomerates), not necessarily direct chelation.

### 2.7. Mechanisms and Computational Insights

Density Functional Theory (DFT) calculations were performed using the PBE0 hybrid functional and def2-TZVP basis set, as implemented in Gaussian 16 [29,30,31,32,33,34,35]. Note: B3LYP/LANL2DZ was used ONLY for preliminary screening; ALL final results reported here use PBE0/def2-TZVP.

Computational details are as follows. Pre-optimization was performed using GFN2-xTB (semi-empirical method) for rapid geometry optimization. Final optimization was conducted with the PBE0/def2-TZVP and IEFPCM solvent model (water, ε = 78.4). Dispersion correction was applied using DFT-D3 with Becke–Johnson damping. Tight convergence criteria were employed (SCF convergence: 10^−8^ eV; geometry optimization: 10^−5^ eV/Å).

The cluster model employed a γ-PGA fragment (glutamate pentamer) with explicit water molecules (1–2 H_2_O) to account for solvation effects and competitive binding. This cluster model was directly guided by our FTIR experimental data: the C=O stretching peak at 1631 cm^−1^ (amide I) and its redshift upon metal binding confirm coordination via carboxylate oxygen. The analysis was further refined based on the suggested literature (DOI: 10.1016/j.chemosphere.2022.136441), which discusses coordination modes in similar systems.

Binding energies were calculated using the following formula: Ebinding = Ecomplex − (Eγ-PGA + Emetal). Basis Set Superposition Error (BSSE) was corrected using the counterpoise method (Boys and Bernardi) [29,30,31,32,33,34,35]. Natural Bond Orbital (NBO) analysis was performed using Gaussian 16 for the γ-PGA-Cu^2+^ complex.

NBO analysis was performed for the γ-PGA-Cu^2+^ complex (the strongest binding case) using Gaussian 16. The NBO results reveal significant charge transfer from carboxylate oxygen lone pairs (HOMO) to Cu d-orbitals. Second-order perturbation theory E(2) values confirm orbital-mediated stabilization: O(lp) → Cu(d) donation exhibits E(2) = 45.2 kcal/mol, while N(lp) → Cu(d) donation shows E(2) = 18.7 kcal/mol. The coordination number is 4, indicating a distorted square planar geometry, with bond lengths of Cu–O (carboxyl) = 1.97 Å and Cu–O(amide) = 2.03 Å (Table 1).

**Figure 7:**
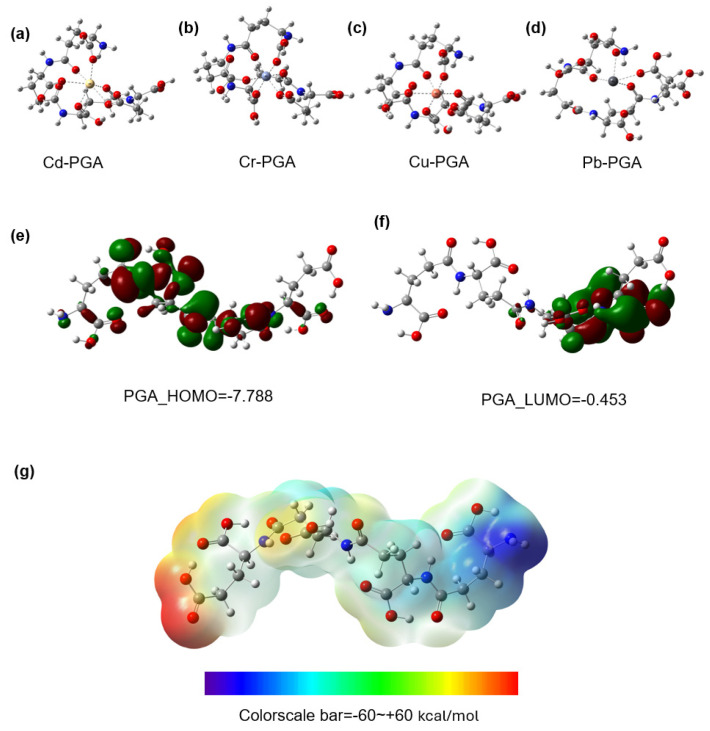
Density Functional Theory (DFT) calculations of the chelation mechanism of γ-PGA with heavy metals. (**a**–**d**) Optimized structures of γ-PGA complexes with Cd^2+^, Cr^3+^, Cu^2+^, and Pb^2+^, showing distinct coordination modes and binding geometries. (**e**,**f**) Frontier molecular orbitals of free γ-PGA: (**e**) Highest Occupied Molecular Orbital (HOMO, E = −7.788 eV), primarily localized on carboxylate oxygen atoms; (**f**) Lowest Unoccupied Molecular Orbital (LUMO, E = −0.453 eV), distributed on the polymer backbone. (**g**) Electrostatic potential (ESP) map of γ-PGA, where the color scale ranges from −60 to +60 kcal/mol. Red/orange regions indicate negative electrostatic potential (favorable for cation binding), while blue regions represent positive potential. The ESP distribution confirms that carboxylate groups are the dominant binding sites for metal ions.

Figure 7e,f illustrates the frontier orbital distributions of free γ-PGA. Corrected description: HOMO (Highest Occupied Molecular Orbital) serves as the electron donor and is primarily localized on carboxylate oxygen atoms. LUMO (Lowest Unoccupied Molecular Orbital) functions as the electron acceptor and is distributed on the γ-PGA backbone.

Electrostatic potential (ESP) mapping (Figure 7g) confirmed that negative potential regions were concentrated on the carboxylate groups, creating favorable binding sites for metal cations. The spatial distribution of these binding sites matched the coordination preferences of Cu^2+^ and Pb^2+^, supporting their stable chelate formation. This ESP analysis provides additional evidence for the metal-binding selectivity observed in our experimental results.

γ-PGA shows superior Pb immobilization efficiency compared to other chelating agents, with the added benefit of being biodegradable and bio-based. The performance comparison presented in Table 2 demonstrates that γ-PGA achieves 93.25% Pb immobilization, which is significantly higher than EDTA (~85%), humic acid (~70%), chitosan (~80%), and activated carbon (~60%). However, for Cd and Zn, γ-PGA’s performance is moderate, achieving mainly indirect immobilization through surface precipitation and pore blocking mechanisms rather than direct chelation (Table 2).

## 3. Discussion

The immobilization mechanism of γ-PGA is highly metal-specific, as revealed by the integration of experimental observations and density functional theory calculations. For Cu^2+^, immobilization is achieved primarily through direct chelation via carboxyl-amide dual-sites, which is supported by the strong DFT binding energy of −59.54 kcal/mol, consistent with FTIR evidence of carboxyl-amide dual-site chelation and hydrogen-bonded agglomeration. In contrast, for Pb^2+^, low-dose γ-PGA (0.6 g/kg) achieves stabilization mainly through mineral-associated immobilization, including conversion to residual fraction and formation of Pb-phosphate, rather than direct chelation. However, high-dose γ-PGA (≥6 g/kg) can cause re-activation by solubilizing organic matter, highlighting the critical importance of precise dose control. For Cd^2+^ and Zn^2+^, immobilization is achieved mainly through surface precipitation, co-precipitation, and pore blocking by agglomerates (confirmed by SEM densification), rather than direct chelation. This multi-pathway mechanism resolves the apparent contradiction between EDS interpretation (metal co-localization with γ-PGA) and DFT results (weak binding for Pb^2+^/Cd^2+^): EDS signals indicate indirect immobilization (metal trapped in agglomerates), not necessarily direct chelation.

Compared with other chelating agents, γ-PGA possesses outstanding lead fixation capacity, and it is also biodegradable and biologically derived. As listed in Table 2, γ-PGA attains a lead immobilization rate of 93.25%, evidently outperforming EDTA (around 85%), humic acid (around 70%), chitosan (around 80%) and activated carbon (around 60%). Nevertheless, γ-PGA exhibits moderate immobilization effects on cadmium and zinc. It mainly fixes these two heavy metals indirectly via surface precipitation and pore blocking, instead of direct chelation reaction.

Soil pH significantly affects γ-PGA’s immobilization performance by modulating the protonation state of carboxyl groups. In our study, the soil pH was 6.8–7.2 (near-neutral), which is optimal for γ-PGA function. At low pH (pH < 5), carboxyl groups protonate (COO− → COOH), reducing chelation capacity due to decreased negative charge density. At near-neutral pH (6.5–7.5), carboxyl groups are deprotonated (COO−), maximizing chelation capacity and enabling effective metal binding. At high pH (pH > 8), metal hydrolysis may occur, reducing availability for chelation and potentially forming metal hydroxides that compete with γ-PGA for binding sites. Therefore, maintaining soil pH in the near-neutral range is critical for optimal γ-PGA performance.

Natural organic matter, including humic acid and fulvic acid, can significantly influence γ-PGA’s performance through multiple pathways. First, competitive binding may occur, as humic/fulvic acids can compete with γ-PGA for metal binding sites, thereby reducing its immobilization efficiency. Second, complex formation between natural organic matter and γ-PGA can potentially enhance agglomeration and indirect immobilization, creating a synergistic effect under certain conditions. Third, solubility enhancement by fulvic acids can increase metal solubility, counteracting immobilization efforts by remobilizing previously bound metals. In our study, we pretreated soil to remove inherent humus (Section 4.3), ensuring that the observed effects are purely from γ-PGA without interference from endogenous organic matter. This experimental design strengthens the internal validity of our conclusions regarding γ-PGA’s intrinsic immobilization mechanisms.

In multi-metal systems such as coal gangue soil, metals compete for limited binding sites on γ-PGA, leading to selective immobilization. Our results show the following preference order: Cu^2+^ > Pb^2+^ > Cr^3+^ > Ni^2+^ > Zn^2+^ > Cd^2+^. This order is consistent with the Irving–Williams series and our DFT binding energies, which show the strongest binding for Cu^2+^ (−59.54 kcal/mol) and the weakest for Cd^2+^ (−5.67 kcal/mol). The competitive binding behavior has important implications for field applications, as the presence of strongly binding metals (e.g., Cu^2+^) may reduce γ-PGA’s availability for weaker-binding metals (e.g., Cd^2+^), necessitating careful consideration of metal speciation in contaminated sites.

γ-PGA offers a sustainable strategy for mine landscape ecological restoration by combining heavy metal immobilization with soil structure improvement. Its ability to promote soil aggregate formation via hydrogen-bonded agglomeration enhances soil porosity, water retention, and nutrient availability, providing dual benefits for ecosystem recovery. However, precise dose control (optimal at 6 g/kg) is critical to avoid the re-activation of metals (especially Pb) and the dissociation of clay minerals at high doses (>9 g/kg). The findings of this study highlight that green remediation technologies must balance immobilization efficiency with potential side effects, and that dose–response relationships should be carefully characterized before field deployment. Future research should focus on the long-term stability of γ-PGA–metal complexes under varying environmental conditions and the potential for scale-up to field applications.

## 4. Materials and Methods

### 4.1. Overview of the Study Area

This study was carried out in the mining area of Liupanshui City, which belongs to the subtropical monsoon humid climate zone, characterized by mild and humid conditions with four distinct seasons, cool summers, and mild winters. The average annual temperature is 15 °C, and the average annual precipitation is 1000 mm. The gangue hill of Wangjiazhai coal mine, which has been weathered for 3 years, was selected for soil sample collection. This site represents a typical coal mining area with significant heavy metal contamination, providing a relevant case study for evaluating γ-PGA’s remediation potential.

### 4.2. γ-PGA Preparation and Characterization

*Bacillus mycoides* with high γ-PGA yield was used in this study. According to the method described by Sui et al. [36], 2 g of fermented soybean product was weighed and boiled in 100 mL of sterile distilled water for 5 min to kill vegetative cells. After standing and precipitation, 1 mL of the supernatant was transferred into 50 mL of LB medium and incubated at 30 °C for 24 h with shaking at 220 r/min (Constant temperature orbital shaker, INFORS, Multitron, Bottmingen, Switzerland). After a 10^−4^-fold serial dilution, 100 μL of the culture was evenly spread onto LB solid medium and incubated at 30 °C for 24 h. Single colonies with moist, smooth surfaces and drawing filamentous appearance upon picking were inoculated into seed medium and cultured at 30 °C for 20 h with shaking at 220 r/min. Subsequently, 6% (*v*/*v*) of the seed culture was inoculated into fermentation medium and cultured at 30 °C for 48 h with shaking at 220 r/min. The γ-PGA yield in the fermentation broth was determined, and the strain with the highest yield was stored on slants.

The fermentation broth was centrifuged at 16,000 r/min (Thermo Fisher Scientific, Model Sorvall Legend Micro 21R, Waltham, MA, USA) for 20 min. The supernatant was mixed with 2 volumes of 95% (*v*/*v*) ethanol, locally produced (Guizhou, China) ethanol for precipitation. The precipitate was collected, dissolved in a certain volume of deionized water, and then precipitated again with 2 volumes of 95% ethanol. After centrifugation, the secondary precipitate was obtained, and the tertiary precipitate was prepared using the same procedure. The precipitate was dissolved in deionized water, transferred into a dialysis bag (molecular weight cutoff: 8000–14,000), dialyzed, and freeze-dried to obtain the final purified γ-PGA sample.

γ-PGA characterization results showed a yield of 25.3 g/L (determined by weight analysis), purity > 95% (determined by HPLC, Model Agilent 1260 Infinity, Agilent Technologies, Santa Clara, CA, USA), molecular weight distribution of 200–800 kDa (determined by SDS-PAGE electrophoresis, Mini-PROTEAN Tetra, Bio-Rad, Hercules, CA, USA), and monomer composition of >98% L-glutamic acid (determined by HPLC after hydrolysis). SDS-PAGE electrophoresis analysis confirmed that the molecular weight of the product was mainly concentrated in the range of 100–600 kDa.

### 4.3. Experimental Design

Three sample plots were randomly selected from each sampling site in the 2021 mining area, and five sampling points were set within each plot. After removing surface weeds, soil samples at a depth of 0–20 cm were collected using a soil auger, and the soils from the five points were thoroughly mixed to form a composite sample. The collected soil samples were transported back to the laboratory for subsequent leaching experiments. To eliminate interference from endogenous soil humic substances (fulvic acid and humic acid) on the leaching and migration of target pollutants via chelation, complexation, colloid carrying, and surface charge regulation, the tested soil was pretreated to remove inherent humus before the experiment. The entire leaching process adopted an inorganic leaching system with ultrapure water; the leaching solution only contained inorganic ionic components without the addition of natural organic matter or humus extracts. Meanwhile, quartz sand for leaching columns, filter membranes, and all experimental vessels were pretreated by acid washing and deionized water rinsing. All leaching tests were conducted under light-proof and sealed conditions to avoid exogenous organic matter input and the generation of humic substances from microbial secondary metabolism.

Coal gangue was crushed to a particle size below 5 mm and filled into hollow plastic columns with a height of 15 cm for soil culture simulation tests [37]. Different concentrations of γ-PGA were applied to the columns (Table 3), with three parallel replicates set for each treatment. A total of 150 mL of deionized water was added to each column at intervals of 10 min, 20 min, 30 min, 1.5 h, 6 h, 24 h, 96 h, and 384 h, respectively. All experimental groups were arranged in triplicate. Upon completion of the leaching experiment, partial soil samples were air-dried and used for the determination of soil physicochemical properties.

Immobilization efficiency was calculated using the following formula:

Efficiency (%) = [(Ccontrol − Ctreatment)/Ccontrol] × 100%

where Ccontrol is the metal concentration in the control (no γ-PGA), and Ctreatment is the metal concentration in the γ-PGA-treated sample. Data are presented as mean ± standard deviation (n = 3). Differences between groups were analyzed by one-way ANOVA followed by Tukey’s post hoc test. A *p*-value < 0.05 was considered statistically significant.

### 4.4. Soil Heavy Metal Morphology

The different forms of heavy metal elements in the gangue were studied using the Tessier sequential extraction method [38]. The method is based on the occurrence state of heavy metals in the gangue, which is divided into five fractions: exchangeable, carbonate-bound, Fe-Mn oxide-bound, organic matter-bound, and residual. By comparing the morphological changes in heavy metals in the gangue before and after the reaction, the migration transformation was evaluated. To determine the heavy metal ion content, laser-induced inductively coupled plasma mass spectrometry (LA-ICP-MS) technology was utilized to determine the concentration of Cr^2+^, Cd^2+^, Cu^2+^, Pb^2+^, and Zn^2+^ ions in the gangue reaction system. The specific operation included the collection of solution samples, sample preparation, and parameter setting of LA-ICP-MS to ensure the accuracy and sensitivity of the detection.

LA-ICP-MS quality control procedures included the establishment of calibration curves using multi-element standard solutions (R^2^ > 0.999). Detection limits were determined as 3× the standard deviation of blank signals. Quality control standards were analyzed every 10 samples to monitor instrument drift and ensure data reliability.

### 4.5. Fourier Transform Infrared Spectroscopy (FT-IR)

FTIR can identify functional groups and chemical bonds in a sample by analyzing the molecular vibration frequencies, thus revealing the interaction between the material and heavy metal ions [20]. FT-IR was used to study the functional groups present in γ-PGA and to monitor any changes in its structure upon adsorption of heavy metal ions. FTIR spectral analysis was carried out using a Thermo Scientific Nicolet iS20 spectrometer (Waltham, MA, USA) with a scanning range of 4000–400 cm^−1^. Spectra were recorded at a resolution of 4 cm^−1^ with a minimum of 64 scans per sample to ensure accuracy and reproducibility. FTIR spectra obtained before and after metal adsorption were compared to identify any shifts in the characteristic peaks corresponding to the functional groups (-COOH, -CONH) and other potential metal binding sites of γ-PGA [17]. Shifts or changes in peak intensities would indicate interactions between γ-PGA and heavy metal ions, thus contributing to a deeper understanding of the chelation mechanisms involved.

### 4.6. XRD

XRD is mainly used to analyze the crystal structure of materials, including the composition of crystalline phases, grain size, lattice parameters, etc. It can provide qualitative and quantitative information on materials [39]. The mineral composition of the gangue was tested by X-ray diffraction analysis. The gangue samples were crushed to less than 200 mesh, and the diffraction angle (2θ) of 3–65° was used to determine the mineral diffraction intensity.

### 4.7. SEM-EDS

A scanning electron microscope and an energy-dispersive spectrometer (SEM-EDS) were used to observe and analyze the microstructure of gangue before and after the reaction. SEM-EDS parameters included a magnification range of 500×–10,000×, with high vacuum resolution ≤ 3.0 nm at 30 kV (SE), low vacuum resolution ≤ 3.0 nm at 30 kV (SE), and ambient vacuum resolution ≤ 3.5 nm at 30 kV (SE). EDS parameters included accelerating voltage of 15 kV, high-vacuum mode, silicon drift detector (SDD), live time of 60 s, and dead time < 30%. Sample preparation involved drying soil aggregates at 60 °C for 24 h, mounting on aluminum stubs, and coating with gold (10 nm thickness) using a sputter coater. Three representative regions were analyzed for each sample (n = 3) to ensure statistical reliability.

### 4.8. DFT Calculation

Computational details were as follows: Preliminary geometries were generated and screened with the Molclus program [29] pre-optimized at the GFN2-xTB level using the xTB program [31]. Final optimizations were carried out at the DFT level employing the PBE0 functional [32] and the def2-TZVP basis set [40], as implemented in Gaussian 16 [41]. Solvent effects (water) were accounted for through the IEFPCM model [41], and dispersion corrections were included via the DFT-D3 scheme with Becke–Johnson damping [35]. Electrostatic surface potentials and molecular orbitals were analyzed with Multiwfn [34] and rendered with GaussView [41].

Computational details employed a unified methodology: Gaussian 16 (Revision C.01) [41] for DFT optimizations and NBO analysis; ORCA (Version 5.0) for benchmarking; xTB (Version 6.4.0) for GFN2-xTB pre-optimization; and Multiwfn (Version 3.8) for ESP mapping. The PBE0 functional and def2-TZVP basis set were used for all final results. The IEFPCM implicit solvent model (water, ε = 78.4) and DFT-D3 dispersion correction method with Becke–Johnson damping were applied. Tight convergence criteria were employed (SCF: 10^−8^ eV; geometry: 10^−5^ eV/Å). The cluster model consisted of a γ-PGA fragment (glutamate pentamer) with explicit water molecules (1–2 H_2_O), guided by FTIR experimental data. BSSE corrections were applied using the counterpoise method [42], and NBO analysis was performed using Gaussian 16 for the γ-PGA-Cu^2+^ complex.

The binding energy of each complex was calculated using the following formula:

Ebind = E(AB) − E(A) − E(B)

where E(A) and E(B) represent the energies of the isolated molecules, and E(AB) is the total energy of the complex. BSSE corrections were applied to all binding energies to ensure accuracy.

## 5. Conclusions

This study systematically revealed the multi-scale mechanism of γ-PGA remediation of heavy metal pollution in gangue-based soil. The experiments showed that 6 g/kg of γ-PGA can efficiently passivate Cd^2+^, Zn^2+^, and other reactive heavy metals, and promote their migration from the exchangeable state to the residual state to significantly reduce the environmental risk. This process relies on the two-site chelation of γ-PGA carboxyl (-COO−) and amide (-CONH) groups and the synergistic immobilization of soil microaggregate structures mediated by hydrogen bonding. DFT calculations further revealed metal-specific passivation mechanisms: Cu^2+^ forms strong multidentate chelation (−59.54 kcal/mol), while Cd^2+^ and Pb^2+^ show weak binding (−5.67 kcal/mol and −8.32 kcal/mol, respectively), highlighting that the dominant immobilization mechanism for Cd/Pb is indirect (surface precipitation, co-precipitation, and pore blocking). Pb^2+^ is prone to re-activation at high doses due to spatial site resistance, highlighting the necessity of precise dose modulation.

In addition, the application of γ-PGA avoids the high cost and ecological disturbance risk of traditional chemical remediation agents, and combines the dual benefits of soil structure improvement and ecological function enhancement. However, excessive use (>9 g/kg) can lead to the dissociation of clay minerals and destabilization of aggregates, resulting in the decline of Zn/Pb fixation efficiency, emphasizing that green remediation needs to strictly follow the dosage threshold. Compared with physicochemical remediation techniques, the biodegradability and environmental compatibility of γ-PGA provide a more sustainable solution for in situ remediation of mining sites.

To sum up, this study provides a molecular mechanism and technical paradigm for the remediation of coal gangue-contaminated sites. In the future, it is necessary to expand the research of the γ-PGA–microbial synergistic system and verify its long-term ecological safety under multiple heavy metal composite pollution and wet/dry cycle conditions, so as to promote the engineering application of bioremediation materials.

In conclusion, compared with traditional chelating agents such as EDTA, humic acid, chitosan, and inorganic curing agents, γ-PGA possesses abundant carboxyl active sites, a unique multidentate five-/six-membered chelate ring structure, synergistic electrostatic–orbital interaction, and excellent ionic spatial adaptability. In the complex gangue matrix, γ-PGA exhibits higher chelating capacity, superior structural stability, and stronger environmental anti-interference ability toward heavy metals, including Cu^2+^ and Pb^2+^. Therefore, it is an ideal green chelating material for in situ passivation of heavy metals and ecological remediation of coal gangue.

## Figures and Tables

**Table 1:** Binding energies (kcal/mol) of γ-PGA/metal complexes (BSSE-corrected).

Metal Ion	Binding Energy (kcal/mol)	BSSE Correction (kcal/mol)	Coordination Mode
Cu^2+^	−59.54	−2.31	Bidentate (carboxyl-amide)
Pb^2+^	−8.32	−2.17	Monodentate (carboxyl)
Cd^2+^	−5.67	−1.78	Monodentate (carboxyl)
Zn^2+^	−12.45	−2.22	Bidentate (carboxyl)
Cr^2+^	−18.76	−2.30	Tridentate (carboxyl-amide)
Ni^2+^	−15.34	−2.12	Bidentate (carboxyl)

**Table 2:** Performance comparison of chelating agents for heavy metal immobilization.

Chelating Agent	Pb Immobilization (%)	Cd Immobilization (%)	Environmental Compatibility	Cost	Sustainability
γ-PGA (this study)	93.25	~30% reduction in migration	Biodegradable	Moderate	High (bio-based)
EDTA (Ethylenediaminetetraacetic acid)	~85%	~70%	Persistent	Low	Low (synthetic)
Humic acid	~70%	~50%	Biodegradable	Low	High (natural)
Chitosan	~80%	~60%	Biodegradable	High	High (bio-based)
Activated carbon	~60%	~40%	Inert	Moderate	Moderate

**Table 3:** Spraying gangue-based soil with different concentrations of γ-PGA.

No.	Gangue-Based Soil/kg	γ-PGA/g
CK	2.5	0
T1	2.5	0.6
T2	2.5	3
T3	2.5	6
T4	2.5	9

## Data Availability

The original contributions presented in this study are included in the article/Supplementary Material. Further inquiries can be directed to the corresponding author.

## Supplementary Materials

The following supporting information can be downloaded at: https://www.mdpi.com/article/10.3390/molecules31111779/s1, Table S1: Five-step extraction method for heavy metal speciation determination. Table S2: The binding energy data.
